# Presence of the *Anopheles culicifacies* complex species A in southeast Iran

**DOI:** 10.1186/s41182-025-00683-y

**Published:** 2025-01-14

**Authors:** Seyed Massoud Madjdzadeh, Nahid Behi, Mohammad Amin Gorouhi, Arsalan Amirkafi, Mohammad Ali Oshaghi

**Affiliations:** 1 Department of Biology, Faculty of Sciences, Shahid Bahonar University of Kerman, Kerman, Iran; 2 Department of Vector Biology and Control, School of Public Health, Kerman University of Medical Sciences, Kerman, Iran; 3 Research Center of Tropical and Infectious Diseases, Kerman University of Medical Sciences, Kerman, Iran; 4 Department of Vector Biology and Control of Diseases, School of Public Health, Tehran University of Medical Sciences, Tehran, Iran

**Keywords:** *Anopheles culicifacies*, Allele-specific PCR, Sequencing PCR, 28S-D3 rDNA, COII, Iran

## Abstract

**Background:**

The *Anopheles culicifacies* complex is one of the most important malaria vectors in Southeast Asia and Southeastern Iran. Although the sibling species within this complex are morphologically indistinguishable, they differ significantly in their disease transmission potential, blood-feeding behaviour, and other biological traits. Cytogenetic and chromosomal studies have identified five sibling species within this complex: A, B, C, D, and E. Understanding the species composition and distribution of this complex is crucial for malaria control strategies.

**Objectives:**

This study aimed to identify the sibling species of the *An. culicifacies* complex in Qaleh Ganj County, Kerman Province, Southeastern Iran. Specifically, the study sought to determine the presence of species A, which is known to be a primary vector of malaria in the region.

**Methods:**

We employed allele-specific PCR and sequencing PCR techniques to identify the sibling species. DNA was extracted from mosquito specimens, and the D3 region of the 28S rDNA gene and a segment of the COII gene from the mitochondrial genome (mtDNA) were targeted for amplification and sequencing.

**Results:**

Data analysis revealed a positive correlation between *An. culicifacies s.l.* specimens and altitude, with most specimens collected from mountainous areas. Both allele-specific PCR and sequencing PCR confirmed the presence of species A in the study areas of Kerman Province.

**Conclusions:**

Given that species A is a primary malaria vector, the findings of this study provide valuable insights for guiding malaria control strategies in Southeastern Iran. Further studies are recommended to assess the vector competence and ecological dynamics of other species within the *An. culicifacies* complex in the region.

## Introduction

In 2022, the World Health Organization (WHO) reported 249 million new malaria cases globally, surpassing the malaria elimination plan's target by 5 million cases [1]. Despite significant progress in Iran's malaria elimination efforts, which led to the eradication of the disease in many high-risk regions, the country still recorded 1439 cases in 2022, including locally transmitted ones. Alarmingly, 2023 saw a several-fold increase in cases compared to the previous year, despite four consecutive years (2018–2021) without any indigenous cases. The provinces most affected in Iran include Sistan and Baluchistan, Hormozgan, and Kerman [2]. Seven Anopheles species are implicated as vectors or proven carriers of malaria in the country: *Anopheles dthali, An. maculipennis s.s., An. stephensi, An. superpictus s.l., An. sacharovi, An. fluviatilis s.l.,* and *An. culicifacies s.l.,* all of which have been reported in various regions of Iran [3–6].

*Anopheles culicifacies *sensu lato Giles 1901 is widely distributed from East Asia, including Cambodia, Vietnam, China, Thailand, and Myanmar, to Central Asia, encompassing Nepal, Bangladesh, and Sri Lanka. Its range extends westward to Afghanistan, Pakistan, Iran, Yemen, and North Africa, including Ethiopia. This mosquito species is the primary vector of malaria in India, responsible for about 60–70% of malaria cases in the country [7]. In Iran, *An. culicifacies* is one of the most significant malaria vectors, particularly in the southeast [8–10].

*Anopheles culicifacies* is recognized as a complex of five sibling species, provisionally designated as A, B, C, D, and E [7, 11]. Laboratory and epidemiological studies, combined with cytological identification, have shown that species A, C, D, and E are vectors of malaria in India, while species B is a poor non-vector [7]. In addition, these sibling species exhibit distinct biological characteristics, such as biting activity, host feeding preferences, and insecticide susceptibility, all of which are crucial for malaria control strategies [12, 13]. Understanding the distribution of vector and non-vector species within this complex is essential for developing targeted control programs [14].

Reliable diagnostic methods are needed to differentiate vector species from non-vectors within the *An. culicifacies* complex. Morphological characteristics alone have proven insufficient for identifying the members of this complex. Polytene chromosome examination can distinguish four members of the complex, where species E is absent, but this technique requires mosquitoes in the half-gravid stage, limiting its application to only a small portion of adult collections and excluding immature stages [15]. Moreover, species E identification requires both polytene chromosome examination and mitotic chromosome analysis of F1 males [11].

DNA-based methods have become the standard for identifying closely related or morphologically indistinguishable species. Mitochondrial DNA has been extensively used for the identification of sibling species across various taxa, including mosquitoes [16–19]. In addition, different regions of nuclear ribosomal DNA (rDNA) have proven to be effective molecular markers for developing diagnostic assays to distinguish members of species complexes [5, 20–22]. A polymerase chain reaction (PCR) assay based on the D3 domain (D3–PCR) of 28S rDNA and a PCR-restriction fragment length polymorphism (PCR–RFLP) assay targeting the ITS2 region of rDNA have been developed for identifying members of the *An. culicifacies* complex [23]. However, these assays can only differentiate species A and D from species B, C, and E. In a study, two allele-specific PCR assays (AD–PCR and BCE–PCR) were performed based on sequence differences in the mitochondrial cytochrome oxidase II (COII) subunit [7]. The AD–PCR assay distinguishes species A and D, while the BCE–PCR assay differentiates species B, C, and E. By combining the D3–PCR/ITS2-RsaI assay with either the AD–PCR or BCE–PCR assays, it is possible to accurately identify all members of this species complex, including distinguishing the non-vector species B from the vector species A, C, D, and E.

*Anopheles culicifacies* has shown a wide altitudinal distribution across its range, raising the question of whether its occurrence is influenced by environmental factors, such as altitude. Previous studies in other regions have suggested that the distribution of malaria vectors, can vary with altitude, which may affect malaria transmission risk [24, 25]. Understanding the relationship between the presence of *An. culicifacies s.l.* and altitude is critical for predicting vector distribution and guiding vector control strategies, particularly in geographically diverse regions like Qaleh Ganj County, Iran. This study includes an analysis of the correlation between altitude and the occurrence of *An. culicifacies s.l.* to provide insights into its potential environmental preferences.

In the present study, we aimed to use COII and D3 (28S rDNA) sequences to identify all members of the *An. culicifacies* complex in Southeastern Iran. This study is the first of its kind in this region, where we aim to clarify the distribution and species composition of the *An. culicifacies* complex, which will be essential for vector control and malaria eradication strategies in Iran.

## Materials and methods

### Study area

**Fig. 1:**
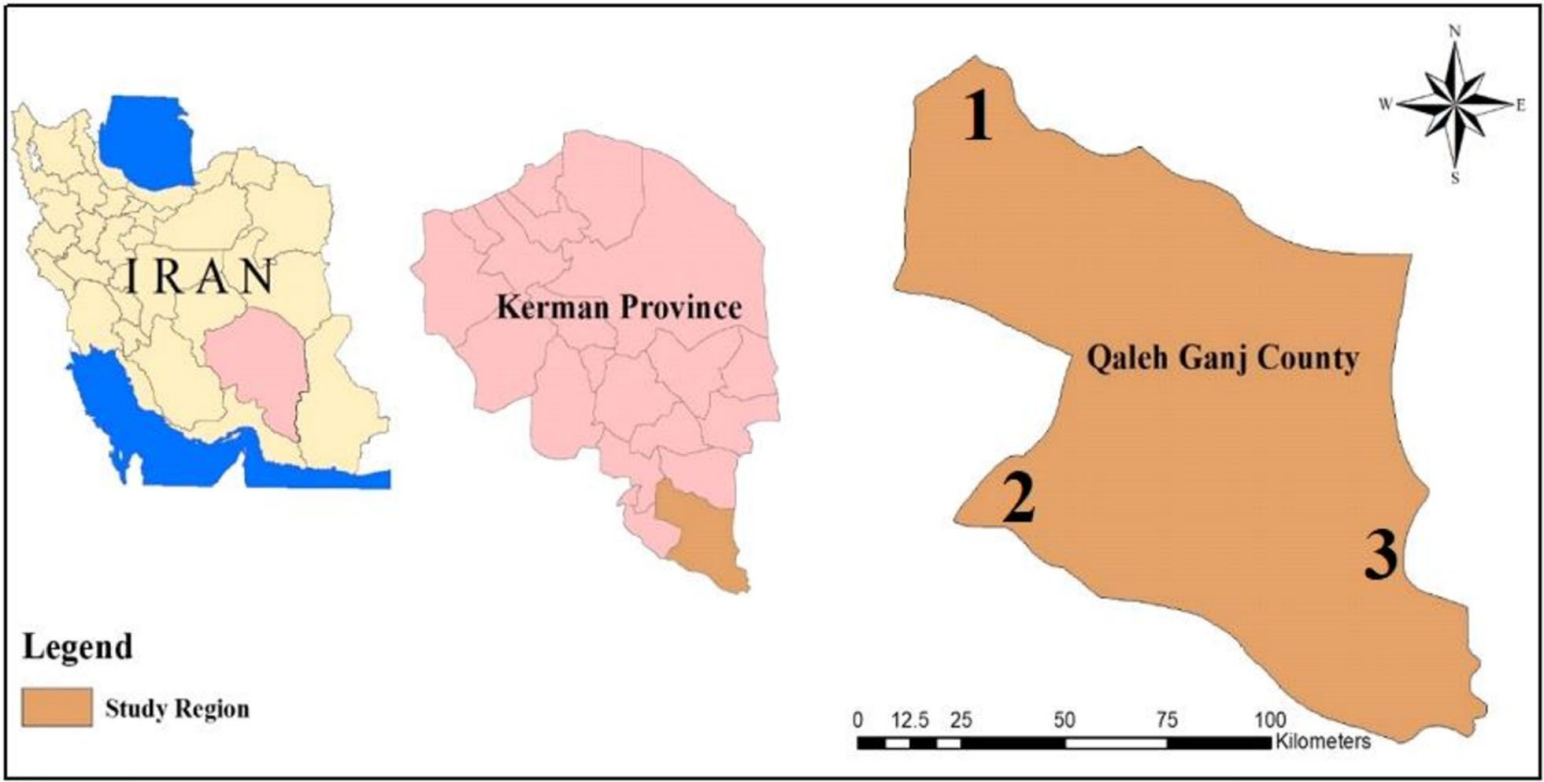
Sampling areas of *Anopheles* mosquitoes in Qaleh Ganj, Kerman Province, southern Iran, 2018. 1: Galou, 2: Shahkahan, 3: Rameshk districts

This study was conducted from April to December 2018 in Qaleh Ganj County, located in the southern part of Kerman province, Iran. Qaleh Ganj lies at a latitude of 27° 32ʹ N and longitude of 57° 50ʹ E, with a population of over 75,000 as of 2016 (Fig. 1). The region is classified as a malarious area, where *An. culicifacies* s.l. is the predominant species [26]. The geographical coordinates and altitudes of the sampling sites were recorded using a Global Positioning System (GPS) device (Garmin 76CS), and these locations were mapped using ArcGIS software, version 10.8 (Redlands, CA). Sampling sites encompassed diverse altitudinal zones, ranging from plains to foothills and mountainous areas, with altitudes between 442 and 1,026 m above sea level.

### Sampling and data collection

Larvae of *Anopheles* mosquitoes were collected monthly from April to December 2018 using the standard dipping technique recommended by the WHO [27]. Sampling occurred in various geographical areas, including Shahkahan, Rameshk, and Galoo districts (Table 1).

**Table 1:** Geographical characterization of the study area in Qaleh Ganj County

Location	Latitude	Longitude	Average Altitude (m)	Topography	Ecotype	Number of collected A*n. culicifacies* s.l
Shahkahan	26˚ 58ʹ 11ʺ N	57˚ 57ʹ 13ʺ E	1026	Mountainous	Elevated village with palm groves	255
Rameshk	26˚ 50ʹ 14ʺ N	58˚48ʹ 37ʺ E	666	Foothill	Mound village with palm groves	44
Galoo	27˚ 47ʹ 28ʺ N	57˚ 57ʹ 03ʺ E	442	Plain	Flat village with farms	11

The collected larvae were transported alive to the Biology and Vector Control Department at Kerman University of Medical Sciences, where they were reared until emergence as adults. Adult mosquitoes were identified to the species level using a standard taxonomic key [28] and stored at – 20 °C until DNA extraction. The presence of *An. culicifacies s.l.* across different altitudinal zones was recorded, and its correlation with altitude was assessed using Pearson’s correlation coefficient.

### DNA extraction

Genomic DNA was individually extracted from the mosquitoes using an animal DNA extraction kit (Dena Zist, Tehran, Iran) according to the manufacturer’s instructions. The extracted DNA was resuspended in 100 µl of TE (Tris–EDTA) buffer and stored at 4 °C for further analysis.

### PCR assays and sequence analysis

For species identification, we followed the protocol described by Goswami et al. [7]. Morphologically identified *An. culicifacies s.l.* specimens were first assessed using the D3–PCR assay [29], which differentiates sibling species into two primary groups: A/D and B/C/E. Specimens classified into the A/D group by D3–PCR were further analysed using the AD–PCR assay to distinguish species A from D. Similarly, those identified as belonging to the B/C/E group were subjected to the BCE–multiplex PCR assay to differentiate species B, C, and E.

The D3 domain of 28S rDNA was amplified using universal primers D3A (5ʹ-GAC CCG TCT TGA AAC ACG GA-3ʹ) and D3B (5ʹ-TCG GAA GGA ACC AGC TAC TA-3ʹ) following the protocol of Goswami et al. [7, 30]. The AD–PCR assay employed three primers (ADF: 5ʹ-CTA ATC GAT ATT TAT TAC AC-3ʹ, ADR: 5ʹ-TTA CTC CTA AAG AAG GC-3ʹ, and DF: 5ʹ-TTA GAG TTT GAT TCT TAC-3ʹ) to distinguish species A from D. The BCE–PCR assay used four primers (BCEF: 5ʹ-AAA TTA TTT GAA CAG TAT TG-3ʹ, BCR: 5ʹ-TTA TTT ATT GGT AAA ACA AC-3ʹ, CR: 5ʹ-AGG AGT ATT AAT TTC GTC T-3ʹ, and ER: 5ʹ-GTA AGA ATC AAA TTC TAA G-3ʹ) to differentiate species B, C, and E.

The amplification conditions for D3–PCR, AD–PCR, and BCE–PCR assays were performed as described in Goswami et al. [7]. A subset of final PCR products was sequenced bidirectionally using the same primers employed for PCR amplification. Sequences were compared using Clustal Omega (https://www.ebi.ac.uk/jdispatcher/msa/clustalo) and verified against GenBank database reference sequences (AJ 519492 for species A, AJ518810 for species B, AJ519493 for species C, AJ519494 for species D, and AJ534646 for species E) using BLAST (https://blast.ncbi.nlm.nih.gov/Blast.cgi). The absence of stop codons in the translated sequences was confirmed using Expasy tools (https://web.expasy.org/translate/) to ensure their accuracy.

## Results

A total of 1042 specimens were collected, with 310 (29.75%) identified as *Anopheles culicifacies s.l.* Our analysis revealed a significant positive correlation between the occurrence of *An. culicifacies s.l*. and altitude (*r* = 0.93). Most specimens (82.26%) were collected from mountainous areas (Shahkahan) at altitudes of 500–1026 m. These findings indicate that *An. culicifacies s.l.* populations were more abundant in higher-altitude regions compared to lower-altitude areas (Table 1).

Based on the D3–PCR assay, samples producing PCR products of 382 bp and 313 bp are classified as belonging to the A/D group, whereas samples with fragments of 385 bp and 133 bp are classified as belonging to the B/C/E group. In the second stage, AD–PCR results indicate that samples with a single 359 bp band belong to species A, while those showing both 359 bp and 166 bp bands are classified as species D. In the final stage, BCE–multiplex PCR is used to differentiate species B, C, and E. If bands of 178 bp and 248 bp are observed, the sample is identified as species E; bands of 95 bp and 248 bp indicate species C; and a single 248 bp band confirms species B.

**Fig. 2:**
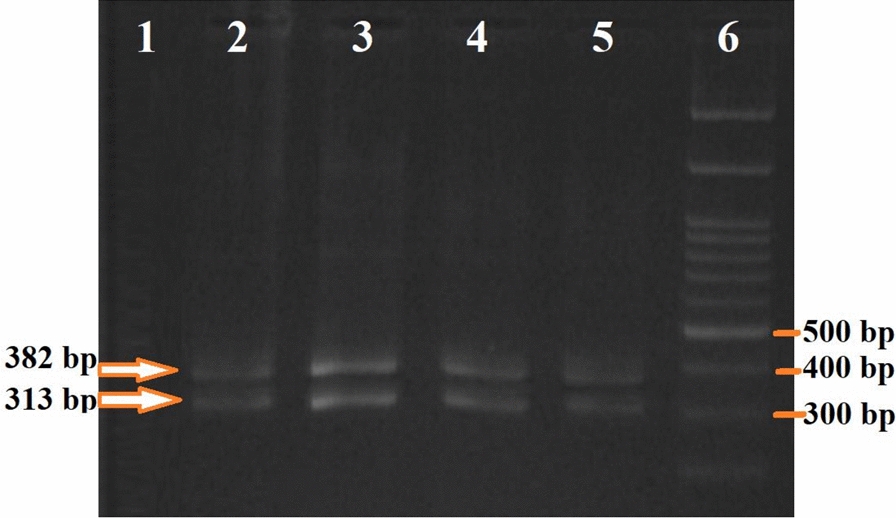
Results of the D3–PCR assay of 28S rDNA using D3A and D3B primers [7], showing two bands at 382 and 313 bp, characteristic of species A/D within the *An. culicifacies* complex in southern Iran. Lane 1: negative control (double-distilled water); Lanes 2–5: samples from this study; Lane 6: 100 bp molecular weight marker (Sinn Clone, Iran)

**Fig. 3:**
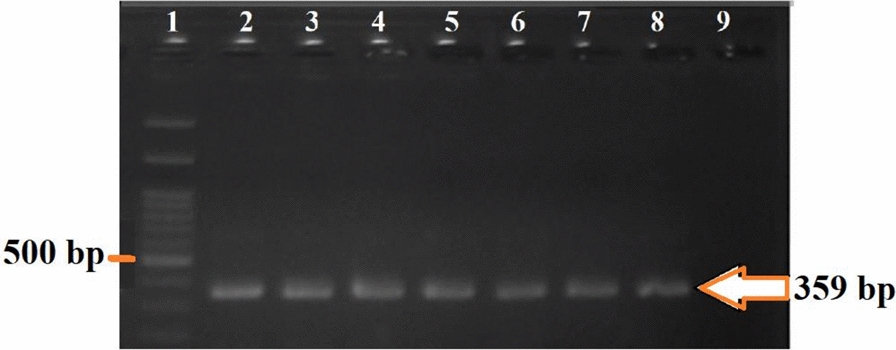
Results of the multiplex AD–PCR assay using specific primers ADR, ADF, and DF, which produced a single 359 bp band characteristic of species *A* of the *An. culicifacies* complex in southern Iran. Lane 1: 100 bp molecular weight marker (Sinn Clone, Iran); Lanes 2–8: samples from this study; Lane 9: negative control (double-distilled water)

In this study, all specimens tested yielded products of 382 bp and 313 bp in the D3–PCR assay, indicating they belong to the A/D group (Fig. 2). According to the results of AS–PCR targeting the COII gene, all specimens were identified as sibling species *A* of *An. culicifacies*, as specific primers ADR, ADF, and DF produced a single 359 bp band, which is characteristic of species A (Fig. 3).

**Fig. 4:**
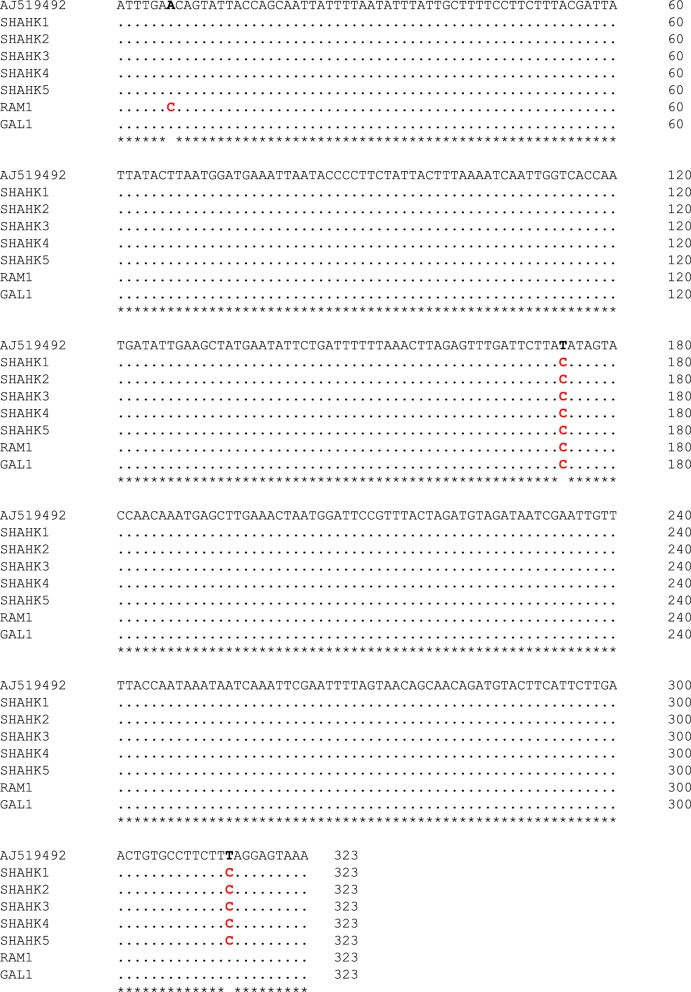
Comparison of a portion (323 bp) of the mitochondrial cytochrome oxidase subunit II (COII) gene sequence from the samples in this study (1–7) with the corresponding sequence of *An. culicifacies* from India (Top row, GenBank ID: AJ519492).: identical to the top row, *: identical position

**Fig. 5:**
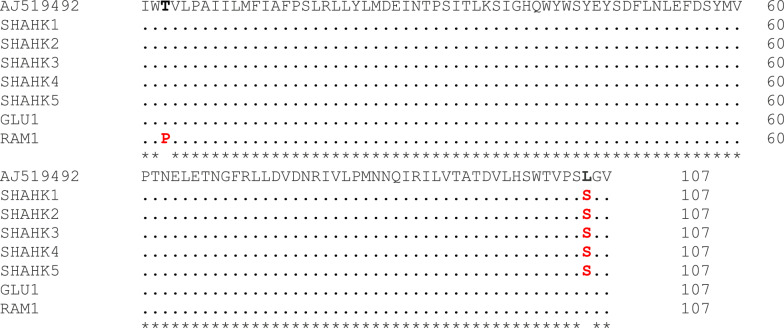
Comparison of a portion (107 AA) of the mitochondrial cytochrome oxidase subunit II (COII) gene sequence from the samples in this study (1–7) with the corresponding sequence of *An. culicifacies* from India (Top row, GenBank ID: AJ519492). Dots mean identical to the top row, *: identical position, gap: non-identical

The sequencing results were compared with sequences available in GenBank, confirming that our samples belong to species A, showing 99.4% similarity to the Indian strain of species A (accession number AJ519492). Sequence analysis revealed three haplotypes (I, II, III) within the samples collected from the study area. Haplotype I (five out of seven specimens) was the most prevalent. Across the 323 bp region analyzed, only three mutations were found: two transitions and one non-informative transversion (Fig. 4). However, two of the three mutations were nonsynonymous, resulting in changes to the amino acid sequence of the COII gene (Fig. 5). Both DNA and amino acid sequence analyses revealed that specimens from the plain (Galoo) and foothill (Rameshk) regions were more similar to the Indian strain than those from the mountainous (Shahkahan) region. The sequence data obtained in this study have been submitted to the GenBank database under accession numbers PQ411020–PQ411026.

## Discussion

This study represents the first molecular identification of *Anopheles culicifacies* sibling species in Qaleh Ganj County, southeast Kerman Province, Iran. Using allele-specific PCR (AS–PCR) and sequencing, we confirmed the presence of sibling species A in the study area. This finding is significant, as species A is a known primary malaria vector, playing a pivotal role in local malaria transmission dynamics. Our results align with earlier reports from Sistan and Baluchistan Province that also identified species A using polytene chromosome banding and PCR–RFLP [30, 31]. The identification of species A in this study expands its known geographic range in Iran, underscoring the need for continuous monitoring of its distribution.

Globally, the identification of *An. culicifacies* sibling species has advanced significantly through various molecular methods. In India, DNA hybridization and AS–PCR assays targeting the D3 domain of the 28S rDNA gene have proven effective for differentiating sibling species [32]. Similarly, multiplex BCE–PCR assays targeting the ITS2 and COII regions have provided reliable differentiation among sibling species A/D and B/C/E [7]. Recent phylogenetic analyses, using both nuclear and mitochondrial markers, have further delineated the complex into distinct clades, revealing geographically associated groupings [32]. The application of these advanced techniques underscores the utility of molecular tools in understanding the evolutionary relationships and distribution of sibling species.

In this study, the application of allele-specific PCR targeting the D3 domain of 28S rDNA and BCE–PCR allowed for precise identification of sibling species A of the *Anopheles culicifacies* complex. This molecular approach not only corroborates the utility of these methods for species differentiation but also highlights the importance of integrating genetic tools with morphological identification in routine entomological surveillance. While these methods are highly effective, they require validation across diverse ecological contexts to ensure robustness and reproducibility.

In addition to allele-specific techniques, other molecular methods such as restriction fragment length polymorphism (RFLP) have been previously used for identifying species within the *Anopheles culicifacies* complex. PCR–RFLP assays targeting regions such as the ITS2 rDNA have been successful in differentiating species A and D from species B, C, and E; however, they are limited in their ability to differentiate all members of the complex. These methods require restriction enzyme digestion, a labour-intensive and costly process, and can sometimes yield ambiguous results due to the presence of overlapping bands or insufficient resolution.

In contrast, allele-specific techniques offer several advantages. By targeting specific DNA sequences unique to each species, these methods enable more precise and reliable species identification, particularly in complex species groups. Allele-specific PCR methods also eliminate the need for time-consuming enzymatic treatments and complicated protocols, making them both cost-effective and easier to scale for large studies. Furthermore, the use of allele-specific primers ensures greater sensitivity and specificity, leading to higher accuracy in species identification, even in mixed samples.

While traditional methods such as RFLP still have their place, the simplicity, accuracy, and efficiency of allele-specific techniques make them a more practical choice for studies involving species complexes like *An. culicifacies*. By reducing costs, labor, and the likelihood of errors, allele-specific techniques represent a promising tool for enhancing surveillance and control strategies for malaria vectors in complex ecological regions.

The observed positive correlation between altitude and the abundance of *An. culicifacies s.l.* underscores the significant role of environmental factors in shaping the distribution of malaria vectors. Higher altitudes may provide optimal conditions such as cooler temperatures, higher humidity, and specific vegetation types that enhance the survival and reproduction of this species. Factors like water temperature, pH, depth, and the type of water bodies (e.g., clear, turbid, or shaded) are critical in defining suitable larval habitats [24, 25]. Vegetation and proximity to human or animal habitations further influence habitat selection, as demonstrated in other vector species like *An. gambia s.s., An. arabiensis, An. funestus*, *An. Demeilloni*, *An. pharoensis, An. cinereus,* and *An. christyi*, which exhibit altitude-specific preferences [24, 25].

The adaptability of *An. culicifacies s.l.* to elevated habitats, as suggested by this study, has critical implications for malaria transmission. This ecological flexibility may allow it to exploit regions with favourable environmental conditions, increasing malaria risk in highland areas, where cooler temperatures and specific breeding habitats prevail. Targeted interventions, such as habitat modification and larval source management, should prioritize these regions to mitigate transmission.

Species A’s role as a primary malaria vector necessitates focused vector control strategies tailored to its ecological and behavioural characteristics. Understanding how factors like vegetation cover, shaded breeding sites, and reduced human disturbance contribute to its abundance in mountainous regions will be essential for designing effective interventions. Future research should further investigate the specific ecological drivers of *An. culicifacies* s.l. distribution to enhance targeted malaria control efforts.

Despite the advances in molecular identification, gaps remain in understanding the ecological and genetic diversity of *An. culicifacies*. For example, while previous studies have demonstrated the utility of mitochondrial and nuclear markers for sibling species identification, discrepancies in marker sensitivity and specificity across geographic regions highlight the need for further validation and standardization. In addition, more comprehensive phylogeographic studies are required to uncover potential cryptic species or genetic variants within the complex that may influence vectorial capacity and insecticide resistance.

Finally, our findings emphasize the need for expanding molecular surveillance to other regions of Iran to assess the full distribution of *An. culicifacies* sibling species. Such studies are critical for informing regional vector control strategies and understanding the broader implications of species distribution shifts, particularly in the context of environmental changes and malaria elimination efforts.

## Conclusion

This study provides valuable insights into the distribution, breeding behaviour, and molecular identification of *An. culicifacies* sibling species in Iran, with significant implications for malaria vector management. Our findings highlight the importance of altitude as a key factor influencing the species' ecological preferences and breeding behaviour. Future research should focus on understanding the adaptive behaviours and genetic diversity of *An. culicifacies* sibling species, as well as their ecological requirements, to develop more effective, evidence-based vector control interventions.

## Data Availability

Sequence data that support the findings of this study have been deposited in the GenBank database with the primary accession numbers: PQ411020–PQ411026.
